# The relationship between dose and serotonin transporter occupancy of antidepressants—a systematic review

**DOI:** 10.1038/s41380-021-01285-w

**Published:** 2021-09-21

**Authors:** Anders Sørensen, Henricus G. Ruhé, Klaus Munkholm

**Affiliations:** 1 Nordic Cochrane Centre, Rigshospitalet, Copenhagen, Denmark; 2 Department of Psychiatry, Radboudumc, Nijmegen, The Netherlands; 3 Donders Institute for Brain, Cognition and Behavior, Radboud University, Nijmegen, The Netherlands; 4 Centre for Evidence-Based Medicine Odense (CEBMO) and Cochrane Denmark, Department of Clinical Research, University of Southern Denmark, Odense, Denmark; 5 Open Patient data Exploratory Network (OPEN), Odense University Hospital, Odense, Denmark

**Keywords:** Biological techniques, Depression, Biochemistry

## Abstract

Brain imaging techniques enable the visualization of serotonin transporter (SERT) occupancy as a measure of the proportion of SERT blocked by an antidepressant at a given dose. We aimed to systematically review the evidence on the relationship between antidepressant dose and SERT occupancy. We searched PubMed and Embase (last search 20 May 2021) for human in vivo, within-subject PET, or SPECT studies measuring SERT occupancy at any dose of any antidepressant with highly selective radioligands ([^11^C]-DASB, [^123^I]-ADAM, and [^11^C]-MADAM). We summarized and visualized the dose-occupancy relationship for antidepressants across studies, overlaying the plots with a curve based on predicted values of a standard 2-parameter Michaelis–Menten model fitted using the observed data. We included seventeen studies of 10 different SSRIs, SNRIs, and serotonin modulators comprising a total of 294 participants, involving 309 unique occupancy measures. Overall, following the Michaelis–Menten equation, SERT occupancy increased with a higher dose in a hyperbolic relationship, with occupancy increasing rapidly at lower doses and reaching a plateau at approximately 80% at the usual minimum recommended dose. All the studies were small, only a few investigated the same antidepressant, dose, and brain region, and few reported information on factors that may influence SERT occupancy. The hyperbolic dose-occupancy relationship may provide mechanistic insight of relevance to the limited clinical benefit of dose-escalation in antidepressant treatment and the potential emergence of withdrawal symptoms. The evidence is limited by non-transparent reporting, lack of standardized methods, small sample sizes, and short treatment duration. Future studies should standardize the imaging and reporting procedures, measure occupancy at lower antidepressant doses, and investigate the moderators of the dose-occupancy relationship.

## Introduction

Many antidepressants are defined by their high affinity for the serotonin transporter (SERT). Brain imaging studies using positron emission tomography (PET) and single photon emission computed tomography (SPECT) techniques have enabled the in vivo visualization of neurotransmitter receptor occupancy, and thus of the proportion of SERT blocked, at a given dose of a drug. By using radioligands that bind to the available SERT receptors, PET and SPECT techniques provide an estimate of the expression of SERT in a particular brain region, usually given as the non-displaceable binding potential (BP_ND_)—the ratio of the specifically bound radioligand to that of non-displaceable radioligand. When antidepressants with an affinity for SERT are administered, the SERT availability for binding of the radioligand decreases, depending on the binding of the antidepressant to SERT. By visualizing the binding potential in the drug free state and subsequently after administration of an antidepressant, PET and SPECT imaging can provide an estimate of the antidepressant occupancy of SERT.

As one postulated working mechanism of many antidepressants is serotonin reuptake inhibition via blockade of SERT [1], PET and SPECT imaging studies may potentially provide mechanistic insight into important clinical aspects of antidepressant treatment relevant to both the effectiveness of the treatment but also symptoms that may arise during tapering or discontinuation of the treatment. The efficacy of antidepressant treatment for depression is modest compared with placebo [2, 3] and many patients may need additional treatment or treatment adjustments. Common treatment strategies in patients with depression for whom antidepressant treatment has not been effective include increasing the antidepressant dose or switching to a different antidepressant; such strategies may, however, also largely be ineffective [4–7]. Although a relation between SERT occupancy and clinical improvement in depressive symptoms has not been shown [1], knowledge about the relationship between antidepressant dose and SERT occupancy, as well as overlapping actions on SERT between different antidepressants, may be able to provide some mechanistic insight into the apparently limited effectiveness of the above treatment strategies. Approximately half of the patients stopping or reducing the dose of antidepressants experience withdrawal symptoms [8], which, among others, may include flu-like symptoms, anxiety, emotional lability, lowering of mood, and irritability [9, 10]. As one postulated mechanism underlying antidepressant withdrawal symptoms is a rapid decrease in SERT occupancy arising when antidepressants are tapered or stopped [11, 12], a clearer understanding of the SERT occupancy at specific, especially lower, doses may provide essential mechanistic information for understanding the occurrence of withdrawal symptoms. Given the paucity of evidence for specific tapering regimens [13, 14], this could perhaps offer insights into better approaches to mitigate withdrawal symptoms. The potential utility of the dose/occupancy relationship in guiding tapering to mitigate withdrawal symptoms has received recent interest by multiple groups [11, 12, 15], and was considered in a recent Cochrane review on approaches to antidepressant discontinuation [14] and in a recent iteration of a major guideline on the management of patients with mood disorders [16].

While several individual studies have investigated the relationship between dose and SERT occupancy of antidepressants [17–19], this evidence has not been systematically reviewed, which we, therefore, aimed to do. Our primary objective was to determine the relationship between the dose and SERT occupancy measured with highly selective radioligands for antidepressants. In studies that repeated measurements of SERT occupancy over time after discontinuation, we additionally aimed to determine the SERT occupancy decline rate and relate it to the plasma concentration decline rate.

## Methods

We conducted a systematic review of in vivo PET and SPECT studies that measured SERT occupancy of antidepressants in humans using a within-subject design. We registered a protocol at the Open Science Framework before undertaking the review, which can be accessed at: https://osf.io/b6hau/. We reported the review according to the PRISMA guidelines [20].

### Search strategy and selection criteria

Studies investigating SERT occupancy at any given dose of any approved antidepressant administered orally in humans were eligible. We included studies using the ligands [^11^C]-DASB, [^123^I]-ADAM, and [^11^C]-MADAM, which all have a 1000:1 affinity for SERT over the dopamine transporter (DAT) and the norepinephrine transporter (NET) [21, 22]. In addition, these ligands have all been extensively validated via kinetic modeling of arterial input sampling [23–26]. We excluded studies using the ligands [^11^C](+)-McN and [^123^I]-ß-CIT due to their non-selectivity for SERT over other receptors: [^123^I]-ß-CIT has nearly equal affinity for SERT and DAT, while [^11^C](+)-McN is considered “likely selective” with an affinity for SERT over NET of between 10:1 and 100:1 [21]. We considered studies using a within-subject design only, not studies using a between-subject design, as our focus was occupancy, which is optimally calculated in a within-subject design; the study design minimizes variance and is, therefore, more likely than a between-subject design to provide reliable estimates of the relationship between antidepressant dose and occupancy.

We searched PubMed and Embase (last search 20 May 2021). The search terms for Embase were: antidepressant.mp. or exp antidepressant agent/ OR (antidepressant* or SSRI or SNRI or “selective serotonin reuptake inhibitor*“ or TCA or tricyclic* or “serotonin-norepinephrine reuptake inhibitor*“).af. AND exp serotonin transporter/ AND (occupancy or “binding potential” or availabilit* or block* or chang* or inhibit* or binding ratio or reduc* or quantific* or alter*).af. AND (PET.mp. or exp positron emission tomography/ + SPECT.mp. or exp single photon emission computed tomography/). The search strategy for PubMed is available in the study protocol. In addition to electronic searches, we scanned the references of retrieved articles and relevant review articles.

### Study selection process

Titles and abstracts were screened for eligibility by two researchers independently (AS and KM). Full-text versions of potentially eligible titles were retrieved and read by two researchers independently (AS and KM). Reasons for exclusion were noted. Disagreements were resolved by discussion, which, in case of unresolved issues, involved a third researcher.

### Data items and data extraction

Data were extracted by two researchers independently using a standardized and piloted extraction form. We extracted the following data items: author, year, antidepressant name, antidepressant dose, occupancy, brain region, antidepressant plasma- or serum concentration, ED_50_, duration of intake of the antidepressant, time lag between administration of last dose and time of scanning, type of scan, ligand, method used to quantify SERT binding potential, characteristics of the study (availability of a study protocol, assessment of drug adherence, time of day of scan, fasting regimen used), and characteristics of the participants (age, sex, diagnosis, smoking status, alcohol use, and other medications used).

We contacted study authors whenever data was not available in the articles. When study authors did not reply to supply the data, we extracted it from graphs where possible using a web plot digitizer. This was needed in one study [17].

### Data synthesis

We described the study characteristics and presented key study characteristics in tables. We assessed, for each study, several factors that may affect the relationship between antidepressant dose and occupancy. These were specification of dosing regimen (duration, dose, assessment of adherence, time of last intake), standardization of laboratory methods (time of day, fasting regimen, precision of scanning results, lag time between dose administration and scanning), clinical characterization of participants (clinical diagnosis, age, gender, co-medication, smoking, alcohol use), method used to quantify binding potential, use of reference region, and the type of radioligand.

In our protocol, we planned to conduct a meta-analysis of occupancy data for each drug and dose and to report the summary mean occupancy (with 95% confidence interval) by presenting the summary estimates of occupancy as a function of dose in graphs for each drug and brain region, provided the studies were sufficiently similar. However, too few studies investigated the same drug, brain region, and dose using the same ligand, design, and duration of drug-intake to make a meta-analysis meaningful. We, therefore, presented occupancy for each dose, drug, and brain region in tables as means and standard deviations (SDs).

We visualized the dose-occupancy relationship by plotting occupancy against dose for antidepressants that were administered at four or more different doses across studies; if several occupancy measures were available per study, we prioritized, those with the shortest lag time between administration of the last dose of the antidepressant and imaging and those with the longest antidepressant treatment duration. Based on those data, to provide a visual reference for the data points, we fitted a 2-parameter Michaelis–Menten model implemented by the drm R package using the formula

$$f\left( {x,K,V_m} \right) = \frac{{V_mx}}{{K + x}}$$

where V_m_ is the horizontal asymptote (expressing maximum occupancy), x is the drug dose and the parameter K is the dose where the occupancy is halfway between 0 and V_m_ [27]. For these models, the lowest dose and occupancy were fixed at 0 and the maximum dose at the highest dosing in any of the included studies. We then plotted the predicted dose-occupancy curve from the model as an overlay to the data plots of occupancy and dose.

In our protocol, we planned to investigate the time-course of the occupancy decline rate as it relates to the antidepressant plasma concentration decline rate, after taking the last dose, by calculating pooled correlation coefficients from the studies that measured occupancy at several different time points. However, for any drug and dose, no more than one study provided such data, and a meta-analysis was therefore not possible. We, therefore, presented those data narratively instead.

## Results

Our literature search identified 793 abstracts. Screening of titles and abstracts identified 100 records for which we obtained a full-text report. After further removing duplicates (*N* = 16), excluding review articles (*N* = 5), studies not measuring SERT occupancy by antidepressants (*N* = 23), studies administering the drug intravenously (*N* = 4), an animal study (*N* = 1), studies using a between-subjects design (*N* = 4), studies using the non-selective ligands [^11^C](+)-McN or [^123^I]-ß-CIT (*N* = 20), studies of drugs not approved for use in depression or that have been withdrawn (*N* = 8), and studies not providing occupancy expressed as a mean (*N* = 3), a total of 16 articles (reporting on 17 unique studies) were included [17–19, 28–40]. In one article [29], outcomes and methods were elaborated in two other publications [41, 42], which we included accordingly. PRISMA flowchart of study selection process is available in Supplementary Figure 1. Excluded studies, with reasons, are available in Supplementary Table 1.

We contacted the authors of 12 articles to obtain missing data, four of whom were able to provide the requested data [31, 33, 35, 38] (previously unreported data is marked (†) in the tables).

The 17 studies investigated 10 different antidepressants using three different ligands ([^11^C]-DASB (*N* = 11), [^11^C]-MADAM (*N* = 2), and [^123^I]-ADAM (*N* = 4)) and comprised a total of 294 participants (Table 1). In two studies the participants participated in two different scans at different doses after a washout period [30, 33], resulting in a total of 309 unique measurements of occupancy.

**Table 1:** Study characteristics of included studies.

Study	Drug	*N*	Total *N* males	Dose range (mg)	Ligand	Lag (hours)	Duration (days)	Age (mean ± SD years)	Diagnosis	RoI	Ref. model
Meyer et al. (2004) [17]	Cit	18	NA^a^	1–60	DASB	6–13	28	NA^a^	Mix of healthy and MDD^b^	Str, BT, ACC, PFC, mid, BC	Logan
	Ven	18		2.4–225							
	Flu	18		1–60							
	Ser	14		10–200							
	Par	14		5–60							
Klein et al. (2006) [18]	Cit	10	20	10–20	ADAM	6	1	NA^c^	Healthy	Mid	SRTM
	Esc	15		5–20							
Lundberg et al. (2007) [19]	Cit	8	16	20	MADAM	6	1	NA	Healthy	ACC, FC, TC, ins, hip, put, rap	SRTM
	Esc	8		10							
Baldinger et al. (2014) [31]	Cit	9	6	20	DASB	6	1 + 25	42.3 ± 7.8	MDD	Accu, ins, amy, cau, put, tha, str, mid	MRTM2
	Esc	10		10							
Klein et al. (2007) [36]	Cit	9	9	20	ADAM	6 + 54	10	28 ± 3	Healthy	Mid	SRTM
	Esc	6		10							
Smith et al. (2011) [38]	Cit	7	4	20–40	DASB	NA	56–70	65 ± 5	MDD	Str, tha	Logan, MRTM2
Houle et al. (2000) [34]	Cit	3	NA	40	DASB	3	1	NA	Healthy	Str, mid, tha	NA
Herold et al. (2006) [37]	Cit	13	11	10	ADAM	6–7	7	NA	MDD	Mid	Logan
Kim et al. (2017) [35]	Esc	12	12	5–30	DASB	3 + 24 + 46	1	23 ± 2.7	Healthy	Put, DRN, cau, tha	MRTM2
Arakawa et al. (2016) [30]	Esc	8	8	10–20	DASB	4 + 24 + 48	1	29.1 ± 4.6	Healthy	Tha	SRTM
	Ser	4		50							
	Par	4		20							
Catafau et al. (2006) [32]	Par	9	NA^d^	20	ADAM	NA	39	NA	MDD	Mid, tha, str	Tissue ratio method
Takano et al. (2006a) [39]	Flv	6	6	50	DASB	5 + 26 + 53	1	24.3 ± 4.8	Healthy	FC, tha, str, hip, amy	MRTM2
Takano et al. (2006b) [40]	Dul	15	15	5–60	DASB	6 + 25 + 49 + 53 + 78	1 + 7	24.1 ± 2.4	Healthy	Tha	MRTM2
Abanades et al. (2011) [28]	Dul	10	10	20	DASB	6, 4	1 + 4	40.2 ± 11	Healthy	Mid, str, tha	SRTM
Areberg et al. (2012) [41]	Vor	35	35	2.5–20	DASB	7	13	NA^a^	Healthy	Rap	SRTM
Areberg et al. (2012) [41, 42]	Vor	11	11	2.5–60	MADAM	7	1 + 9	NA^a^	Healthy	Rap	SRTM
Frankle et al. (2018) [33]	Des	15	8	25–150	DASB	24^e^	3	27 ± 9	Healthy	Mid, tha, amy, str	SRTM

*N* number of participants, *lag (hours)* time lag in hours between drug administration and scanning, *SD* standard deviation, *RoI* brain region of interest, *Ref. model* reference model for quantifying binding potential, *cit* citalopram, *ven* venlafaxine, *flu* fluoxetine, *ser* sertraline, *par* paroxetine, *esc* escitalopram, *flv* fluvoxamine, *dul* duloxetine, *vor* vortioxetine, *des* desvenlafaxine, *NA* not applicable, *MDD* major depressive disorder, *str* striatum, *BT* bilateral thalamus, *ACC* anterior cingulate cortex, *PFC* prefrontal cortex, *mid* midbrain, *BC* bilateral cuneus, *FC* frontal cortex, *TC* temporal cortex, *ins* insula, *hip* hippocampus, *put* putamen, *rap* raphe nuclei, *accu* accumbens, *amy* amygdala, *tha* thalamus, *cau* caudate, *DRN* dorsal raphe nucleus, *SRTM* simplified reference tissue model, *MRTM-2* multilinear reference tissue model 2.

^a^Information on sex and age provided only for 77 of 82 participants in Meyer 2004 (33 females, 44 males, mean age (SD) 35 (9), and only for both groups combined in Areberg 2012 (46 males, mean age 28 years (21–41).

^b^Healthy participants received low doses, unhealthy participants received high doses.

^c^Mean age data includes four dropouts (26.8 years for all 29 participants).

^d^Participant characteristics include one dropout (6 males, 4 females, mean age (SD) 36 (10.8), range 20–53).

^e^Unique to this review

The designs of the studies investigated were highly heterogeneous, involving 16 different regions of interest, 11 different durations of drug-intake, 15 different periods of time lag between administration of last dose and time of scanning, two different health statuses (depressed patients or healthy controls), and four different modeling-approaches. An arterial input function was used in one study [33]. The included studies also varied in duration of drug-intake after the (drug-free) baseline scan until imaging of the drug-occupancy, with six studies administering the drug just once and 10 studies using a repeated dose regimen (ranging from 4 days to 10 weeks).

All included studies controlled for adherence to the antidepressant used in the study by measuring plasma- or serum concentrations. Nine of the included studies provided no information on whether participants were taking other drugs and stated no such restrictions to study entry. Eight studies excluded participants taking other possibly interacting medications (e.g., other psychotropic drugs or substances with high affinity for SERT) [19, 31, 32, 37–40, 42]. A fasting regimen was mentioned in one study [35]: requiring a minimum of 4 h fasting prior to imaging. Two studies mentioned having standardized the time of day of drug administration and scanning [19, 35]. One study reported overall alcohol consumption during the study period [35], and seven studies controlled for present or past alcohol problems or abuse [17, 28, 30, 33, 34, 39, 40]. Five of 17 studies included non-smokers only, while the remaining studies did not mention smoking status [19, 30, 35, 39, 40]. None of the studies provided information on a pre-published study protocol.

SERT occupancy of escitalopram was investigated in 55 participants (six studies) [18, 19, 30, 31, 35, 36], citalopram in 77 participants (eight studies) [17–19, 31, 34, 36–38], vortioxetine in 46 participants (two studies presented in one article) [41], paroxetine in 27 participants (three studies) [17, 30, 32], duloxetine in 25 participants (two studies) [28, 40], sertraline in 18 participants (two studies) [17, 30], venlafaxine in 18 participants (one study) [17], fluoxetine in 18 participants (one study) [17], desvenlafaxine in 8 participants (one study) [33], and fluvoxamine in six participants (one study) [39].

**Fig. 1:**
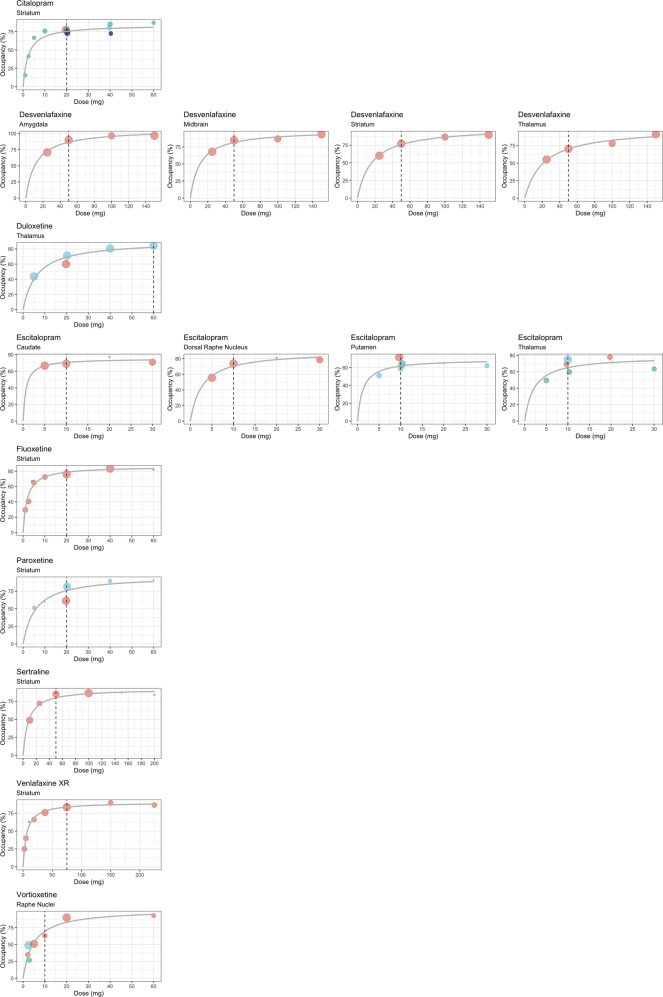
Occupancy and dose relationship for antidepressants administered at four or more doses. The dose-occupancy relationship for antidepressants that were administered at four or more different doses (citalopram, desvenlafaxine, duloxetine, escitalopram, fluoxetine, paroxetine, venlafaxine, and vortioxetine), fitted according to a 2-parameter Michaelis–Menten model as $f\left( {x,K,V_m} \right) = \frac{{V_mx}}{{K + x}}$, where V_m_ is the horizontal asymptote (expressing maximum occupancy), x is the drug dose and the parameter K is the dose where the occupancy is halfway between 0 and V_m_. The parameter estimates V_m_ and K for each model are provided in Supplementary Table 2. For each individual figure, studies are represented by uniquely colored dots; the size of the dots is proportional to the number of occupancy measures. Dashed vertical lines represent the usual minimum recommended dose.

Occupancy at different doses and brain regions is presented in Table 2 as the range of means (SD) for each drug. Four studies calculated and reported the dose corresponding to 50% of maximal occupancy (ED_50_). ED_50_ values in mg were 3·4 for citalopram [17], 9·1 for sertraline [17], 5 for paroxetine [17], 2·7 for fluoxetine [17], 5·8 for venlafaxine [17], 7·9 for duloxetine [40], 8·5 for vortioxetine [42], and 14·4 for desvenlafaxine [33]. Figure 1 illustrates the dose-occupancy relationship for antidepressants that were administered at four or more different doses (citalopram, desvenlafaxine, duloxetine, escitalopram, fluoxetine, paroxetine, venlafaxine, and vortioxetine). The relationship between dose and SERT occupancy was fitted according to a 2-parameter Michaelis–Menten model for all antidepressants separately: occupancy increased hyperbolically with increasing antidepressant dose in the lower dose-range, reaching a plateau at an occupancy of approximately 80% at roughly the usual minimum recommended dose for depression (Fig. 1). The relationship between dose and occupancy appeared largely similar across drugs (Fig. 1). For desvenlafaxine and escitalopram, the relationship between dose and occupancy appeared relatively consistent across brain regions; only for escitalopram, the plateau appeared to be reached at a slightly lower occupancy in the putamen, compared with other brain regions, as shown in Table 2 and Fig. 1. The parameter estimates for the Michaelis–Menten model for each antidepressant and brain region of interest (RoI) are provided in Supplementary Table 2.

**Table 2:** Serotonin transporter occupancy at different antidepressant doses.

Dose in mg	*N*	RoI (number of occupancy measures)	Occupancy (mean ± SD)	Duration of drug-intake (days)
Citalopram				
1 (17)	2	str (2)	16 ± 6%	28
2.5 (17)	2	str (2)	42 ± 1%	28
5 (17)	2	str (2)	67 ± 18%	28
10 (17, 18, 37)	21	overall	61–76 ± 9%	
		str (3)	76 ± 9%	28
		mid (22)	61–65 ± 10%	7, 1
20 (17–19, 31, 36, 38)	39	overall	64 ± 13–91 ± 5	
		str (17)	74 ± 5–77 ± 10	1, 28
		mid (23)	64 ± 13–86 ± 4	10, 25
		ACC (17)	75 ± 16–80 ± 21	1, 25
		FC (8)	75 ± 14	1
		TC (8)	66 ± 19	1
		ins (17)	71 ± 12–73 ± 9	25, 1
		hip (8)	78 ± 17	1
		put (17)	68 ± 5–76 ± 4	1, 25
		rap (8)	76 ± 9	1
		amy (9)	91 ± 5	25
		cau (9)	80 ± 5	25
		tha (13)	74 ± 7–77 ± 6	56–70, 25
		accu (9)	84 ± 4	25
30 (38)†	1	overall	67–70	
		str (1)	67	56–70
		tha (1)	70	56–70
40 (17, 34, 38)^a^	6	overall	80 ± 5–85 ± 4	
		str (8)	73 ± 6–85 ± 4	56–70, 28
		mid (3)	80 ± 5	1
		tha (5)	80 ± 7–80 ± 5	56–70, 1
60 (17)	2	str (2)	87 ± 6	28
Escitalopram				
5 (18, 35)^a^	9	overall	50 ± 1–67 ± 7	
		mid (5)	60 ± 6	1
		put (4)	51 ± 1	1
		DRN (4)	56 ± 2	1
		cau (4)	67 ± 7	1
		tha (4)	50 ± 1	1
10 (18, 19, 30, 31, 35, 36)	37	overall	59 ± 5–88 ± 9	
		mid (20)	64 ± 6–81 ± 5	1, 10
		ACC (18)	65 ± 11–75 ± 8	1, 25
		FT (8)	67 ± 12	1
		TC (8)	63 ± 23	1
		ins (18)	59 ± 15–69 ± 9	1, 25
		hip (8)	59 ± 23	1
		put (22)	59 ± 5–72 ± 4	1, 25
		rap (8)	69 ± 13	1
		amy (10)	88 ± 9	25
		cau (14)	69 ± 5–78 ± 4	1, 25
		tha (18)	60 ± 3–75 ± 4	1, 25
		str (10)	73 ± 4	25
		accu (10)	81 ± 4	25
		DRN (4)	74 ± 8	25
20 (18, 30, 35)^a^	10	Overall	65–81	
		mid (5)	75 ± 5	1
		put (1)	65	1
		DRN (1)	81	1
		cau (1)	77	1
		tha (5)	72–78 ± 3	1, 1
30 (35)^a^	3	overall	62 ± 3–79 ± 6	
		put (3)	62 ± 3	1
		DRN (3)	79 ± 6	1
		cau (3)	71 ± 11	1
		tha (3)	64 ± 7	1
Sertraline				
10 (17)	3	str (3)	49 ± 13	28
25 (17)	2	str (2)	72 ± 4	28
50 (17, 30)	7	overall	74 ± 6–85 ± 7	1, 28
		str (3)	85 ± 7	28
		tha (4)	74 ± 6	1
100 (17)	4	str (4)	86 ± 3	28
150 (17)	1	str (1)	87	28
200 (17)	1	str (1)	84	28
Paroxetine				
5 (17)	2	str (2)	52 ± 16	28
10 (17)	1	str (1)	60	28
20 (17, 30, 32)	21	overall	45 ± 21–93 ± 8	
		str (17)	61 ± 11–82 ± 10	39, 28
		BT (7)	75 ± 16	28
		ACC (7)	76 ± 15	28
		PFC (7)	80 ± 18	28
		tha (13)	45 ± 21–63 ± 10	1, 39
		mid (16)	66 ± 10–93 ± 8	39, 28
		BC (7)	67 ± 29	28
40 (17)	2	str (2)	90 ± 2	28
60 (17)	1	str (1)	91	28
Fluoxetine				
1 (17)	2	str (2)	30 ± 6	28
2.5 (17)	2	str (2)	41 ± 13	28
4 (17)	1	str (1)	67	28
5 (17)	2	str (2)	65 ± 6	28
10 (17)	2	str (2)	73 ± 1	28
20 (17)	4	overall	69 ± 9–85 ± 9	28
		str (4)	76 ± 8	28
		BT (4)	69 ± 9	28
		ACC (4)	80 ± 14	28
		PFC (4)	85 ± 9	28
		mid (4)	82 ± 9	28
		BC (4)	81 ± 6	28
40 (17)	4	str (4)	83 ± 9	28
60 (17)	1	str (1)	82	28
Fluvoxamine				
50 (39)	6	overall	71 ± 2–76 ± 3	
		FC (6)	75 ± 9	28
		tha (6)	72 ± 4	28
		str (6)	71 ± 2	28
		hip (6)	76 ± 3	28
		amy (6)	72 ± 13	28
Venlafaxine				
2.4 (17)	2	str (2)	25 ± 13	28
5 (17)	2	str (2)	40 ± 1	28
10 (17)	1	str (1)	63	28
18.75 (17)	2	str (2)	66 ± 3	28
37.5 (17)	3	str (3)	76 ± 10	28
75 (17)	4	overall	71 ± 10–92 ± 5	28
		str (4)	84 ± 2	28
		BT (4)	71 ± 10	28
		ACC (4)	85 ± 13	28
		PFC (4)	91 ± 11	28
		mid (4)	91 ± 8	28
		BC (4)	92 ± 5	28
150 (17)	2	str (2)	90 ± 1	28
225 (17)	2	str (2)	87 ± 4	28
Duloxetine				
5 (40)	3	tha (3)	44 ± 9	1
20 (28, 40)	13	overall	71 ± 5–85 ± 4	
		tha (13)	71 ± 5–74 ± 7	1, 4
		mid (10)	85 ± 4	4
		str (10)	75 ± 8	4
40 (40)	3	tha (3)	81 ± 5	1
60 (40)	3	tha (3)	85 ± 3	7
Vortioxetine				
2.5 (41, 42)	16	RN (16)	35 ± 10–49 ± 12	9, 13
5 (41)	11	RN (11)	51 ± 10	13
10 (42)	4	RN (4)	63 ± 23	9
20 (41)	12	RN (12)	90 ± 6	13
60 (42)	3	RN (3)	93 ± 9	9
Desvenlafaxine				
25 (33)	4	overall	55 ± 5–71 ± 13	
		mid (4)	68 ± 8	3
		tha (4)	55 ± 5	3
		amy (4)	71 ± 13	3
		str (4)	60 ± 7	3
50 (33)	4	overall	70 ± 8–90 ± 9	
		mid (4)	85 ± 7	3
		tha (4)	70 ± 8	3
		amy (4)	90 ± 9	3
		str (4)	77 ± 7	3
100 (33)	3	overall	78 ± 2–96 ± 5	
		mid (3)	87 ± 6	3
		tha (3)	78 ± 2	3
		amy (3)	96 ± 5	3
		str (3)	87 ± 1	3
150 (33)	4	overall	90 ± 3–97 ± 4	
		mid (4)	94 ± 6	3
		tha (4)	91 ± 10	3
		amy (4)	97 ± 4	3
		str (4)	90 ± 3	3

Where a dose was only investigated in one study, the mean (SD) occupancy of that individual study is presented; where a dose was investigated in multiple studies, the range of means (SD) from those studies is presented. Duration of drug-intake for the individual studies is presented in the same order as the occupancy range.

*N* number of participants, *RoI* brain region of interest (number of occupancy measures in the different regions), *SD* standard deviation, *str* striatum, *mid* midbrain, *ACC* anterior cingulate cortex, *FC* frontal cortex, *TC* temporal cortex, *ins* insula, *hip* hippocampus, *put* putamen, *rap* raphe nuclei, *amy* amygdala, *cau* caudate, *tha* thalamus, *accu* accumbens, *DRN* dorsal raphe nucleus, *BC* bilateral cuneus.

^a^Full occupancy data is unique to this review.

**Fig. 2:**
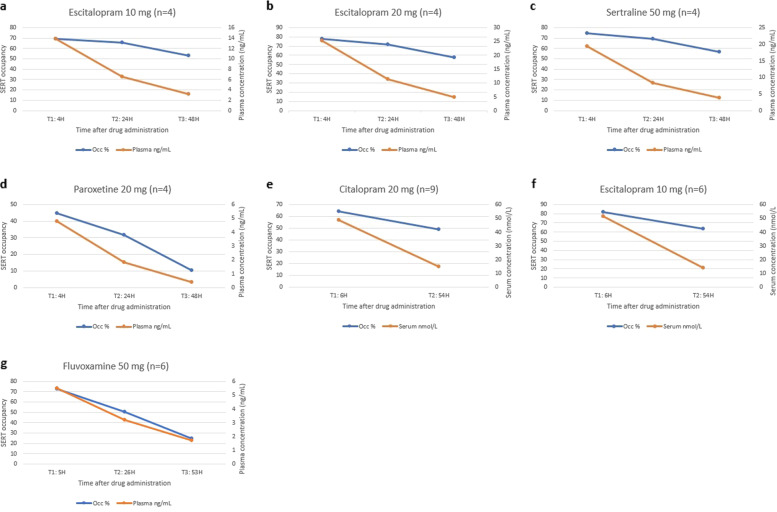
Time-course of occupancy and plasma/serum concentration. The relationship between serotonin transporter occupancy and plasma/serum concentration as it decreases over time after dose administration of escitalopram, citalopram, sertraline, paroxetine, and fluvoxamine. mg milligram, N number of participants, SERT Serotonin transporter, H hours after drug administration, Occ serotonin transporter occupancy, ng/mL nanograms per milliliter, nmol/L nanomoles per litre.

Five studies provided data on the time-course of SERT occupancy as it decreases over time after a single dose [30, 35, 39, 40] or repeated (7–10 days) [36, 40] administration of antidepressants in healthy volunteers. Three of these studies compared the decline rates of occupancy and plasma concentration (Fig. 2) [30, 36, 39]. For escitalopram [30], citalopram [36], and sertraline [30], but not paroxetine [30] and fluvoxamine [39], SERT occupancy appeared to decrease at a slower rate than the plasma concentration (Fig. 2).

Three studies measured occupancy after both single and repeated dosing in the same subjects [28, 31, 41], all of which showed increased occupancy after repeated dosing compared with a single dose. For duloxetine at 20 mg, occupancy increased from 65% to 78% after four days [28]. For citalopram at 20 mg and escitalopram at 10 mg, occupancy increased from 73% and 74% to 80% and 77%, respectively, after 25 days [31]. For vortioxetine at 2·5, 10, and 60 mg, occupancy increased from 27%, 44%, and 70%, to 35%, 63%, and 93%, respectively, after nine days [41].

## Discussion

For this first systematic review of the evidence of the relationship between dose and SERT occupancy of antidepressants we identified 17 studies investigating 10 different SSRIs, SNRIs and serotonin modulators in a total of 294 participants, comprising 309 unique measurements of SERT occupancy with highly specific ligands. Overall, occupancy increased with higher dose but in a hyperbolic pattern: occupancy increased rapidly at lower doses and reached an apparent plateau at approximately 80%––at the usual minimum recommended dose––modeled by the Michaelis–Menten equation. Generally, the studies were small, only a few studies investigated the same antidepressant at the same dose and in the same RoI, and few studies reported information on relevant factors that may influence drug metabolization and hence bioavailability of antidepressants (e.g. use of potentially interacting drugs, smoking status [43], and alcohol consumption [44]).

The Michaelis–Menten curves we reconstructed based on the findings in individual studies showed that overall, there was no substantial increase of SERT occupancy with SSRI and SNRI doses above the usual minimum recommended doses for depression. This finding is in accordance with one dose-escalation occupancy study of paroxetine using the β-CIT, a ligand not included in the present review, that found no increase in SERT occupancy, nor a clinical effect as a result of paroxetine dose escalation from 20 to 50 mg, despite observed increases in paroxetine serum concentration [45]. It is also in agreement with the observation that there is a limited benefit associated with increasing doses of SSRIs above the lower range of the licensed dose in general, whether using a fixed dosing regimen [4, 5], a flexible dosing regimen [46], or as a second-step strategy in patients not responding to standard dosing [6, 45]. Adverse events, in contrast, may have an ascending dose-response curve [4, 47], presumably due to co-affinity to other receptors. There is no firm evidence that clinical efficacy [1, 17, 45], nor adverse effects are dependent on SERT occupancy; the studies included in the present review that measured the relationship between clinical effect and SERT occupancy did not find significant correlations [17, 32, 37, 38, 48].

Even at, and below, the lowest manufactured dose of the antidepressants we included, there is considerable SERT occupancy. For all drugs except vortioxetine, 50% occupancy occurred at doses quite below half of the lowest manufactured dose. Given this hyperbolic relationship between dose and SERT occupancy, even relatively small dose changes at the lower dose range will have large effects on SERT occupancy and thus presumably on synaptic serotonin levels—with progressively increasing magnitude as the dose decreases linearly towards zero. This finding may particularly have implications for discontinuation and tapering, as a linear tapering regimen, involving stopping at the lowest manufactured dose, or even half of it would correspond to increasingly larger reductions in occupancy, which might be related to the occurrence of withdrawal symptoms. Assuming that withdrawal symptoms are predominantly related to unblocking of SERT, a linear and gradual unblocking of SERT, which has been suggested to mitigate withdrawal symptoms [11, 45], would require a hyperbolic dose reduction regimen, necessitating smaller dose decrements than possible with currently manufactured antidepressants. Eventually, this assumption should be tested in a blinded RCT, where the primary hypothesis would be that discontinuation via hyperbolic tapering should be more successful, with less withdrawal symptoms than stopping via the lowest manufactured dose (or half of it).

Conversely, even large dose reductions above the occupancy plateau appear to be associated with only relatively minor decreases in SERT occupancy, and dose reductions at higher doses may therefore potentially be less likely to result in marked withdrawal symptoms although, similar to the lack of evidence for a relationship with efficacy, there is no unequivocal evidence of a correlation between changes in SERT occupancy and withdrawal symptoms. As SSRIs, SNRIs, and serotonin modulators are not selective to SERT [49–52], dose reductions in the higher dose-range, while not markedly reducing SERT occupancy, could potentially also result in changes in other transmitter systems that could potentially be associated with withdrawal symptoms. However, given that the shape of the receptor occupancy curve is hyperbolic for most dose–response relationships, consequent to the law of mass action [53, 54], hyperbolic dose reduction is likely pharmacologically meaningful regardless of the specific receptor systems contributing to withdrawal symptoms.

The observation that escitalopram, citalopram, and sertraline SERT occupancy appear to decrease at a slower rate compared with the plasma concentration of the drugs may indicate that plasma half-life does not accurately reflect the rate at which SERT occupancy declines. This apparently delayed decrease in SERT occupancy compared with the plasma concentration decline could be speculated to contribute to the delayed withdrawal effects, which have been observed clinically.

Several issues related to the study designs must be taken into consideration when interpreting our findings since these could contribute to the heterogeneity of findings between studies. The included studies that measured occupancy after both single and repeated dosing all found that occupancy increased with longer treatment, the absolute occupancy being in the range of 3 to 23% higher after repeated dosing compared with single dosing [28, 31, 41]. This suggests that occupancy data from single-dose studies may underestimate the occupancy occurring in patients who take the drugs continuously for extended periods. A possible reason for this finding is that after the first administration of an antidepressant these lipophilic drugs dissolve into the entire body, which only becomes saturated after repeated dosing. Additionally, studies varied in the duration between the last dose administration and time of scanning (ranging from three to 24 h), which could underlie some of the heterogeneity of the observed SERT occupancy, although this is expected to cause less variability after longer treatment when a steady-state of the antidepressant plasma concentration will be established. It is also not clear whether our findings are generalizable to all patients on antidepressants, especially for all ages and both sexes, as most included studies investigated healthy individuals in the age range from 25 to 40 years, of whom most were male. Few studies provided precise information on all factors relevant to measuring occupancy, and accurate participant characteristics were sometimes difficult to determine since some studies included dropouts and later excluded participants when reporting age and sex characteristics. Tobacco smoking [43], alcohol consumption [44], fasting [55], and concomitant drug use [56, 57] may influence antidepressant metabolization and thus the bioavailability of the drugs, which, in turn, could affect the relationship between antidepressant dose and occupancy, but information on those factors was not available in most studies. Sample sizes were generally small; five drugs were investigated in less than 20 participants each, and some doses were investigated in just one to three participants. Consideration should also be given to the fact that PET and SPECT are known to detect signals of unspecific radioligand binding, i.e. noise that does not represent target occupancy. Therefore, a reference region assumed devoid of specific SERT binding must be used as control, assuming that signals detected in this brain region do not represent specific binding to SERT. Cerebellum was earlier suggested as the optimal reference region, but later studies revealed that cerebellum is not completely devoid of specific SERT binding. Therefore, the binding potential will be slightly overestimated if this issue is not specifically controlled for, which only one study did [35]. Since all included studies used cerebellum as reference region, this will have resulted in a small and systematic bias. Finally, occupancies in smaller brain regions, like the amygdala, are likely determined with less certainty compared with larger brain regions due to issues of radiotracer reliability in brain regions that are small relative to the resolution of SPECT.

Our study is the first to review the body of evidence of the relationship between dose and SERT occupancy of antidepressants. This not only provides a complete picture of the evidence across many different antidepressants but also allows for a more detailed assessment of the relationship between dose and SERT occupancy by integrating data from different studies for the same antidepressants. Importantly, it also allowed for an assessment of the limitations in the evidence base. In addition to these strengths, our study has several limitations. First, we presented findings from studies using different reference tissue models together; these could influence occupancy measures. However, one study calculated occupancy using three different methods, and found only minor differences between SRTM, MRTM-2, and logan [38], potentially indicating that the reference tissue model does not substantially bias SERT occupancy. Second, comparison of occupancy data from different brain regions is potentially not meaningful, as SERT is not equally distributed throughout the brain [1]. It is therefore possible that the reported occupancy measures do not reflect those areas which are most important for patients, clinically. Whether blocking of SERT in some brain regions is of particular importance regarding treatment effect, adverse events, or withdrawal symptoms remains unresolved. Third, our literature search may have missed some studies that did measure occupancy but used different terms due to inconsistent nomenclature for occupancy and binding potential, especially in the field’s earlier stages. However, our search strategy was created with this in mind, and we systematically scanned the reference lists of all included studies. Fourth, our plot overlays fitted using the Michalis-Menten model did not consider the sample size of individual studies; an overlay plot based on individual patient data would have been preferable, but we did not obtain those data. Along this line, the dose ranges were limited for some drugs, which means that it is uncertain whether the observed plateau for those drugs at approximately 80% occupancy represents the highest possible level of the plateau, e.g. for escitalopram the highest dose was 30 mg and for duloxetine 60 mg. Lastly, in order to investigate differences in occupancy between single- and repeated dosing regimens, which appear to yield different occupancy levels, we synthesized the evidence using both dosing regimens but due to few studies, it was not possible to construct fitted curves stratified by dosing regimen. Along the same line, we included studies regardless of whether co-medication was allowed, which may have contributed to heterogeneity in results between studies; due to the scarcity of data it was not possible to explore the potential effect of co-medication.

### Implications for future research

Our review points to a need for larger occupancy studies of antidepressants administered also at low doses, investigation of moderators of the dose-occupancy relationship, standardization of methods, assessment of associations with clinical effects, and more transparent reporting. Future studies should also measure other transporters (e.g., NET and DAT) than SERT to uncover the full biological effects of the drugs as many antidepressants also act on non-serotonergic systems. As many patients take antidepressants for years, studies should also study patients (before and) after long treatment duration. Finally, the theoretical link between unblocking of SERT and withdrawal symptoms should ideally be investigated directly, for example by measuring occupancy with repeated measurements during a period of dose reduction while recording the occurrence of potential withdrawal symptoms. The feasibility of conducting such studies, however, would be challenged by likely high costs associated with long follow-up, an unknown event rate, and issues associated with performing repeated PET procedures.

### Conclusion

PET and SPECT studies provide a mechanistic background for understanding the limited effect of dose-escalation of antidepressants and for the potential emergence of withdrawal symptoms even with small dose reductions in the lower dose range. The evidence base is limited by few, small studies of short treatment duration and sub-optimal, non-uniform reporting, which should be improved in the future. Such improvements could lead to a better understanding of factors influencing SERT occupancy and the association with treatment efficacy, adverse effects, and withdrawal symptoms after dose-reductions or stopping of antidepressants.

## Supplementary information

Supplementary Information

## Data Availability

All data (including template data collection forms, data extracted from included studies, and data used for all analyses) are available on the Open Science Framework at: 10.17605/OSF.IO/RKYFS.
