# Supplementary material for: The relationship between dose and serotonin transporter occupancy of antidepressants—a systematic review
Source: Mol Psychiatry. 2021 Sep 21;27(1):192–201. doi: 10.1038/s41380-021-01285-w (PMC8960396; doi:10.1038/s41380-021-01285-w)
Supplement: Supplementary file 1 — Supplementary Information [file 41380_2021_1285_MOESM1_ESM.docx]

**Supplementary Information**

**The relationship between dose and serotonin transporter occupancy of antidepressants – a systematic review**

Anders Sørensen MSc,^1,^* Dr Henricus G Ruhé,^2,3^ and Klaus Munkholm, DMSc.^4,5^

^1^ Nordic Cochrane Centre, Rigshospitalet, 7811, Blegdamsvej 9, 2100 Copenhagen, Denmark
^2^ Department of Psychiatry, Radboudumc, Route 966, Reinier Postlaan 4, 6525 GC Nijmegen, the Netherlands

^3^ Donders Institute for Brain, Cognition and Behavior, Radboud University, Kapittelweg 29, 6525 EN Nijmegen, the Netherlands

^4^ Centre for Evidence-Based Medicine Odense (CEBMO) and Cochrane Denmark, Department of Clinical Research, University of Southern Denmark, JB Winsløwsvej 9b, 3^rd^Floor, 5000 Odense, Denmark

^5^ Open Patient data Exploratory Network (OPEN), Odense University Hospital, Odense, Denmark

**Supplementary Figure 1.** PRISMA flowchart of study selection process

**
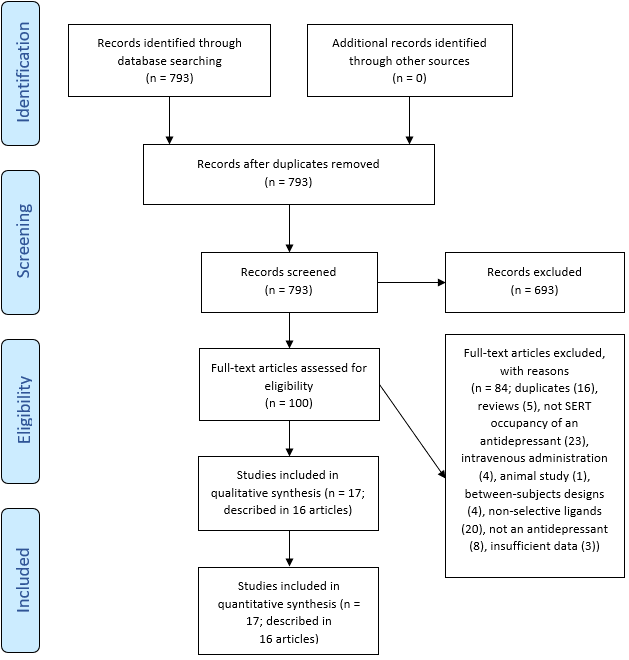
**

**Supplementary Table 1.** List of excluded studies with reasons


**Animal studies (N=1)**

Plisson C., Stehouwer J.S., Voll R.J., Howell L., Votaw J.R., Owens M.J., Goodman M.M., 2007. Synthesis and in vivo evaluation of fluorine-18 and iodine-123 labeled 2beta-carbo(2-fluoroethoxy)-3beta-(4’-((Z)-2-iodoethenyl)phenyl) nortropane as a candidate serotonin transporter imaging agent. J. Med. Chem. 50, 4553–4560. <https://doi.org/10.1021/jm061303s>

**Studies using a between-subjects design (N=4)**

Lundberg J., Tiger M., Landen M., Halldin C., Farde L., 2012. Serotonin transporter occupancy with TCAs and SSRIs: A PET study in patients with major depressive disorder. Int. J. Neuropsychopharmacol. 15, 1167–1172. <https://doi.org/10.1017/S1461145711001945>

Nogami T., Takano H., Arakawa R., Ichimiya T., Fujiwara H., Kimura Y., Kodaka F., Sasaki T., Takahata K., Suzuki M., Nagashima T., Mori T., Shimada H., Fukuda H., Sekine M., Tateno A., Takahashi H., Ito H., Okubo Y., Suhara T., 2013. Occupancy of serotonin and norepinephrine transporter by milnacipran in patients with major depressive disorder: A positron emission tomography study with [11C]DASB and (S,S)-[18F]FMeNER-D2. Int. J. Neuropsychopharmacol. 16, 937–943. <https://doi.org/10.1017/S1461145712001009>

Van De Giessen E., Booij J., 2010. The SPECT tracer [123I]ADAM binds selectively to serotonin transporters: A double-blind, placebo-controlled study in healthy young men. Eur. J. Nucl. Med. Mol. Imaging 37, 1507–1511. <https://doi.org/10.1007/s00259-010-1424-2>

Voineskos A.N., Wilson A.A., Boovariwala A., Sagrati S., Houle S., Rusjan P., Sokolov S., Spencer E.P., Ginovart N., Meyer J.H., 2007. Serotonin transporter occupancy of high-dose selective serotonin reuptake inhibitors during major depressive disorder measured with [11C]DASB positron emission tomography. Psychopharmacology 193, 539–545. <https://doi.org/10.1007/s00213-007-0806-z>

**Duplicates (N=16)**

Akimova E., Savli M., Haeusler D., Fink M., Hahn A., Wadsak W., Mitterhauser M., Lanzenberger R., Kletter K., Kasper S., 2009. Increase of 5-HTT occupancy during escitalopram or citalopram treatment correlates with antidepressant efficacy in major depressive disorder. Eur. Neuropsychopharmacol., 22 ECNP Congress. Istanbul Turkey. 19, S424–S425. <https://doi.org/10.1016/S0924-977X%2809%2970658-3>

Baldinger P., Kranz G.S., Savli M., Wadsak W., Haeusler D., Hahn A., Mitterhauser M., Philippe C., Kasper S., Lanzenberger R., 2013. Regional differences of SERT occupancy in major depression: An in vivo PET study using [11C]DASB. Eur. Neuropsychopharmacol., 2013 ECNP Workshop on Neuropsychopharmacology for Young Scientists in Europe. Nice France. 23, S80–S81.

Frankle G., Robertson B., Maier G., Paris J., Asmonga D., Chen C.-M., May M., Mason N.S., Mathis C.A., Narendran R., 2012. An open-label PET study to evaluate serotonin transporter (SERT) occupancy following escalating doses of Desvenlafaxine (Pristiqs). J. Cereb. Blood Flow Metab., 9th International Symposium on Functional Neuroreceptor Mapping of the Living Brain, NRM 2012. Baltimore, MD United States. 32, S59–S60. <https://doi.org/10.1038/jcbfm.2012.78>

James G.M., Baldinger-Melich P., Philippe C., Kranz G.S., Vanicek T., Hahn A., Gryglewski G., Hienert M., Spies M., Traub-Weidinger T., Mitterhauser M., Wadsak W., Hacker M., Kasper S., Lanzenberger R., 2017. Effects of selective serotonin reuptake inhibitors on interregional relation of serotonin transporter availability in major depression. Front. Human Neurosci. 11, 48. <https://doi.org/10.3389/fnhum.2017.00048>

Kasper, S., Sacher, J., Klein, N., Mossaheb, N., Attarbaschi-Steiner, T., Lanzenberger, R., Spindelegger, C., Asenbaum, S., Holik, A., Dudczak, R., 2009. Differences in the dynamics of serotonin reuptake transporter occupancy may explain superior clinical efficacy of escitalopram versus citalopram. Int Clin Psychopharmacol 24, 119–125. <https://doi.org/10.1097/YIC.0b013e32832a8ec8>

Kim E., Howes O.D., Park J.W., Kim S.N., Shin S.A., Kim B.-H., Turkheimer F.E., Lee Y.-S., Kwon J.S., 2016. Altered serotonin transporter binding potential in patients with obsessive-compulsive disorder under escitalopram treatment: [11C]DASB PET study. Psychol Med 46, 357–366. <https://doi.org/10.1017/S0033291715001865>

Kranz G., Haeusler D., Akimova E., Savli M., Hahn A., Mitterhauser M., Spindelegger C., Wadsak W., Lanzenberger R., Kasper S., 2012a. Serotonin transporter ratio between raphe nuclei and projection areas predicts SSRI treatment response in major depression. Eur. Neuropsychopharmacol., 2012 ECNP Workshop on Neuropsychopharmacology for Young Scientists in Europe. Nice France. 22, S85.

Kranz G., Lanzenberger R., Haeusler D., Philippe C., Savli M., Hahn A., Mitterhauser M., Spindelegger C., Wadsak W., Kasper S., 2012b. Prediction of SSRI treatment response in major depression based on serotonin transporter binding ratios. Eur. Neuropsychopharmacol., 25th European College of Neuropsychopharmacology, ECNP Congress. Vienna Australia. 22, S251.

Kugaya A., Sanacora G., Staley J.K., Malison R.T., Bozkurt A., Khan S., Anand A., Van Dyck C.H., Baldwin R.M., Seibyl J.P., Charney D., Innis R.B., 2004. Brain serotonin transporter availability predicts treatment response to selective serotonin reuptake inhibitors. Biol. Psychiatry 56, 497–502. <https://doi.org/10.1016/j.biopsych.2004.07.001>

Lanzenberger R., Kranz G.S., Haeusler D., Akimova E., Savli M., Hahn A., Mitterhauser M., Spindelegger C., Philippe C., Fink M., Wadsak W., Karanikas G., Kasper S., 2012. Prediction of SSRI treatment response in major depression based on serotonin transporter interplay between median raphe nucleus and projection areas. NeuroImage 63, 874–881. <https://doi.org/10.1016/j.neuroimage.2012.07.023>

Meyer J.H., Wilson A.A., Ginovart N., Goulding V., Hussey D., Hood K., Houle S., 2001. Occupancy of serotonin transporters by paroxetine and citalopram during treatment of depression: A [11C]DASB PET imaging study. Am. J. Psychiatry 158, 1843–1849. <https://doi.org/10.1176/appi.ajp.158.11.1843>

Ruhe H.G., Koster M., Booij J., van Herk M., Veltman D.J., Schene A.H., 2014. Occupancy of serotonin transporters in the amygdala by paroxetine in association with attenuation of left amygdala activation by negative faces in major depressive disorder. Psychiatry Res. Neuroimaging 221, 155–161. <https://doi.org/10.1016/j.pscychresns.2013.12.003>

Ruhe, H.G., Ooteman, W., Booij, J., Michel, M.C., Moeton, M., Baas, F., Schene, A.H., 2009. Serotonin transporter gene promoter polymorphisms modify the association between  paroxetine serotonin transporter occupancy and clinical response in major depressive disorder. Pharmacogenet Genomics 19, 67–76. <https://doi.org/10.1097/FPC.0b013e32831a6a3a>

Savli M., Baldinger P., Haeusler D., Kranz G.S., Philippe C., Spies M., Wadsak W., Mitterhauser M., Kasper S., Lanzenberger R., 2014. Alterations of molecular connectivity in major depression before and during treatment with selective serotonin reuptake inhibitors. Eur. Neuropsychopharmacol., 27th European College of Neuropsychopharmacology, ECNP Congress. Berlin Germany. 24, S403.

Schrantee A., Lucassen P.J., Reneman L., Booij J., 2018. Serotonin transporter occupancy predicts default-mode network connectivity: A spect and resting-state fMRI study. Biol. Psychiatry, 73rd Annual Scientific Convention and Meeting of the Society of Biological Psychiatry, SOBP 2018. United States. 83, S182.

Stenkrona P., Halldin C., Lundberg J., 2013. 5-HTT and 5-HT1A receptor occupancy of the novel substance vortioxetine (Lu AA21004). A PET study in control subjects. Eur. Neuropsychopharmacol. 23, 1190–1198. <https://doi.org/10.1016/j.euroneuro.2013.01.002>

**Studies administering the drug intravenously (N=4)**

Frokjaer V.G., Pinborg L.H., Madsen J., De Nijs R., Svarer C., Wagner A., Knudsen G.M., 2008. Evaluation of the serotonin transporter ligand 123I-ADAM for SPECT studies on humans. J. Nucl. Med. 49, 247–254. <https://doi.org/10.2967/jnumed.107.046102>

Hinz R., Selvaraj S., Murthy N.V., Bhagwagar Z., Taylor M., Cowen P.J., Grasby P.M., 2008. Effects of citalopram infusion on the serotonin transporter binding of [11C]DASB in healthy controls. J. Cereb. Blood Flow Metab. 28, 1478–1490. <https://doi.org/10.1038/jcbfm.2008.41>

Turkheimer, F.E., Selvaraj, S., Hinz, R., Murthy, V., Bhagwagar, Z., Grasby, P., Howes, O., Rosso, L., Bose, S.K., 2012. Quantification of ligand PET studies using a reference region with a displaceable fraction: application to occupancy studies with [(11)C]-DASB as an example. J Cereb Blood Flow Metab 32, 70–80. <https://doi.org/10.1038/jcbfm.2011.108>

Ziebell M., Holm-Hansen S., Thomsen G., Wagner A., Jensen P., Pinborg L.H., Knudsen G.M., 2010. Serotonin transporters in dopamine transporter imaging: A head-to-head comparison of dopamine transporter SPECT radioligands 123I-FP-CIT and 123I-PE2I. J. Nucl. Med. 51, 1885–1891. <https://doi.org/10.2967/jnumed.110.078337>

**Studies using the non-selective ligands [11C](+)-McN or [^123^I]-b-CIT (N=20)**

De Win M.M.L., Habraken J.B.A., Reneman L., Van Den Brink W., Den Heeten G.J., Booij J., 2005. Validation of [123I]beta-CIT SPECT to serotonin transporters in vivo in humans: A double-blind, placebo-controlled, crossover study with the selective serotonin reuptake inhibitor citalopram. Neuropsychopharmacology 30, 996–1005. <https://doi.org/10.1038/sj.npp.1300683>

Kent J.M., Coplan J.D., Lombardo I., Hwang D.-R., Huang Y., Mawlawi O., Van Heertum R.L., Slifstein M., Abi-Dargham A., Gorman J.M., Laruelle M., 2002. Occupancy of brain serotonin transporters during treatment with paroxetine in patients with social phobia: A positron emission tomography study with [11C]McN 5652. Psychopharmacology 164, 341–348. <https://doi.org/10.1007/s00213-002-1218-8>

Kugaya A., Seneca N.M., Snyder P.J., Williams S.A., Malison R.T., Baldwin R.M., Seibyl J.P., Innis R.B., 2003. Changes in human in vivo serotonin and dopamine transporter availabilities during chronic antidepressant administration. Neuropsychopharmacology 28, 413–420.

Marksteiner J., Walch T., Bodner T., Gurka P., Donnemiller E., 2003. Fluoxetine in Alzheimer’s Disease with Severe Obsessive Compulsive Symptoms and a Low Density of Serotonin Transporter Sites. Pharmacopsychiatry 36, 207–209. <https://doi.org/10.1055/s-2003-43051>

Parsey R.V., Kegeles L.S., Hwang D.-R., Simpson N., Abi-Dargham A., Mawlawi O., Slifstein M., Van Heertum R.L., Mann J.J., Laruelle M., 2000. In vivo quantification of brain serotonin transporters in humans using [11C]McN 5652. J. Nucl. Med. 41, 1465–1477.

Pirker, W., Asenbaum, S., Kasper, S., Walter, H., Angelberger, P., Koch, G., Pozzera, A., Deecke, L., Podreka, I., Brucke, T., 1995. beta-CIT SPECT demonstrates blockade of 5HT-uptake sites by citalopram in the human brain in vivo. J Neural Transm Gen Sect 100, 247–256.

Pogarell, O., Poepperl, G., Mulert, C., Hamann, C., Sadowsky, N., Riedel, M., Moeller, H.-J., Hegerl, U., Tatsch, K., 2005. SERT and DAT availabilities under citalopram treatment in obsessive-compulsive disorder (OCD). Eur Neuropsychopharmacol 15, 521–524. <https://doi.org/10.1016/j.euroneuro.2005.01.003>

Rominger A., Cumming P., Brendel M., Xiong G., Zach C., Karch S., Tatsch K., Bartenstein P., la Fougere C., Koch W., Pogarell O., 2015. Altered serotonin and dopamine transporter availabilities in brain of depressed patients upon treatment with escitalopram: A [123I]beta-CIT SPECT study. Eur. Neuropsychopharmacol. 25, 873–881. <https://doi.org/10.1016/j.euroneuro.2014.12.010>

Ruhe H.G., Booij J., Weert H.C.V., Reitsma J.B., Fransen E.J.F., Michel M.C., Schene A.H., 2009. Evidence why paroxetine dose escalation is not effective in major depressive disorder: A randomized controlled trial with assessment of serotonin transporter occupancy. Neuropsychopharmacology 34, 999–1010. <https://doi.org/10.1038/npp.2008.148>

Sassarini J., Krishnadas R., Cavanagh J., Nicol A., Pimlot S., Ferrell W., Lumsden M.A., 2013. Venlafaxine alters cutaneous microvascular perfusion, Beta-CIT binding and BDI scores in flushing postmenopausal women. BJOG Int. J. Obstet. Gynaecol., RCOG World Congress 2013. Liverpool United Kingdom. 120, 565. <https://doi.org/10.1111/1471-0528.12352>

Schrantee A., Lucassen P.J., Booij J., Reneman L., 2018. Serotonin transporter occupancy by the SSRI citalopram predicts default-mode network connectivity. Eur. Neuropsychopharmacol. 28, 1173–1179. <https://doi.org/10.1016/j.euroneuro.2018.07.099>

Shang, Y., Gibbs, M.A., Marek, G.J., Stiger, T., Burstein, A.H., Marek, K., Seibyl, J.P., Rogers, J.F., 2007. Displacement of serotonin and dopamine transporters by venlafaxine extended release capsule at steady state: a [123I]2beta-carbomethoxy-3beta-(4-iodophenyl)-tropane single photon emission computed tomography imaging study. J Clin Psychopharmacol 27, 71–75. <https://doi.org/10.1097/JCP.0b013e31802e0017>

Stengler-Wenzke K., Muller U., Barthel H., Angermeyer M.C., Sabri O., Hesse S., 2006. Serotonin transporter imaging with [123I]beta-CIT SPECT before and after one year of citalopram treatment of obsessive-compulsive disorder. Neuropsychobiology 53, 40–45. <https://doi.org/10.1159/000090702>

Suhara T., Takano A., Sudo Y., Ichimiya T., Inoue M., Yasuno F., Ikoma Y., Okubo Y., 2003. High levels of serotonin transporter occupancy with low-dose clomipramine in comparative occupancy study with fluvoxamine using positron emission tomography. Arch. Gen. Psychiatry 60, 386–391. <https://doi.org/10.1001/archpsyc.60.4.386>

Szabo, Z., Kao, P.F., Scheffel, U., Suehiro, M., Mathews, W.B., Ravert, H.T., Musachio, J.L., Marenco, S., Kim, S.E., Ricaurte, G.A., 1995. Positron emission tomography imaging of serotonin transporters in the human brain using [11C](+)McN5652. Synapse 20, 37–43. <https://doi.org/10.1002/syn.890200107>

Takano A., Suhara T., Sudo Y., Inoue M., Hashimoto K., Zhang M.-R., Ichimiya T., Yasuno F., Suzuki K., 2002. Comparative evaluation of two serotonin transporter ligands in the human brain: [11C](+)McN5652 and [11C]cyanoimipramine. Eur. J. Nucl. Med. Mol. Imaging 29, 1289–1297. <https://doi.org/10.1007/s00259-002-0884-4>

Tammela L.I., Rissanen A., Kuikka J.T., Karhunen L.J., Bergstrom K.A., Repo-Tiihonen E., Naukkarinen H., Vanninen E., Tiihonen J., Uusitupa M., 2003. Treatment improves serotonin transporter binding and reduces binge eating. Psychopharmacology 170, 89–93. <https://doi.org/10.1007/s00213-003-1519-6>

Tauscher J., Pirker W., De Zwaan M., Asenbaum S., Brucke T., Kasper S., 1999. In vivo visualization of serotonin transporters in the human brain during fluoxetine treatment. Eur. Neuropsychopharmacol. 9, 177–179. <https://doi.org/10.1016/S0924-977X%2898%2900013-3>

Zitterl W., Aigner M., Stompe T., Zitterl-Eglseer K., Gutierrez-Lobos K., Wenzel T., Zettinig G., Hornik K., Pirker W., Thau K., 2008. Changes in thalamus-hypothalamus serotonin transporter availability during clomipramine administration in patients with obsessive-compulsive disorder. Neuropsychopharmacology 33, 3126–3134. <https://doi.org/10.1038/npp.2008.35>

Zitterl W., Stompe T., Aigner M., Zitterl-Eglseer K., Ritter K., Zettinig G., Hornik K., Asenbaum S., Pirker W., Thau K., 2009. Diencephalic Serotonin Transporter Availability Predicts Both Transporter Occupancy and Treatment Response to Sertraline in Obsessive-Compulsive Checkers. Biol. Psychiatry 66, 1115–1122. <https://doi.org/10.1016/j.biopsych.2009.07.009>

**Studies of drugs not approved for (or later discontinued as) antidepressants (N=8)**

DeLorenzo C., Lichenstein S., Schaefer K., Dunn J., Marshall R., Organisak L., Kharidia J., Robertson B., Mann J.J., Parsey R.V., 2011. SEP-225289 serotonin and dopamine transporter occupancy: A PET study. J. Nucl. Med. 52, 1150–1155. <https://doi.org/10.2967/jnumed.110.084525>

Park J.-S., Lee J., Meyer J., Ilankumaran P., Han S., Yim D.-S., 2014. Serotonin transporter occupancy of SKL10406 in humans: Comparison of pharmacokineticpharmacodynamic modeling methods for estimation of occupancy parameters. Transl. Clin. Pharmacol. 22, 83–91. <https://doi.org/10.12793/tcp.2014.22.2.83>

Risinger R., Bhagwagar Z., Luo F., Cahir M., Miler L., Mendonza A.E., Meyer J.H., Zheng M., Hayes W., 2014. Evaluation of safety and tolerability, pharmacokinetics, and pharmacodynamics of BMS-820836 in healthy subjects: A placebo-controlled, ascending single-dose study. Psychopharmacology 231, 2299–2310. <https://doi.org/10.1007/s00213-013-3391-3>

Smith J.A.M., Bourdet D.L., Daniels O.T., Ding Y.-S., Gallezot J.-D., Henry S., Kim K.H.S., Kshirsagar S., Martin W.J., Obedencio G.P., Stangeland E., Tsuruda P.R., Williams W., Carson R.E., Patil S.T., 2015. Preclinical to clinical translation of CNS transporter occupancy of TD-9855, a novel norepinephrine and serotonin reuptake inhibitor. Int. J. Neuropsychopharmacol. 18, 1–11. <https://doi.org/10.1093/ijnp/pyu027>

Spies M., James G., Berroteran-Infante N., Ibeschitz H., Unterholzner J., Godbersen M., Gryglewski G., Hienert M., Jungwirth J., Pichler V., Kranz G., Reiter B., Winkler D., Mitterhauser M., Stimpfl T., Hacker M., Kasper S., Lanzenberger R., 2017. Investigating dose dependency of ketamine binding on the serotonin transporter with positron emission tomography. Eur. Neuropsychopharmacol., 30th European College of Neuropsychopharmacology Congress, ECNP 2017. France. 27, S779.

Spies M., James G.M., Berroteran-Infante N., Ibeschitz H., Kranz G.S., Unterholzner J., Godbersen M., Gryglewski G., Hienert M., Jungwirth J., Pichler V., Reiter B., Silberbauer L., Winkler D., Mitterhauser M., Stimpfl T., Hacker M., Kasper S., Lanzenberger R., 2018. Assessment of Ketamine binding of the serotonin transporter in humans with positron emission tomography. Int. J. Neuropsychopharmacol. 21, 145–153. <https://doi.org/10.1093/ijnp/pyx085>

Talbot P.S., Bradley S., Clarke C.P., Babalola K.O., Philipp A.W., Brown G., McMahon A.W., Matthews J.C., 2009. Brain serotonin transporter occupancy by oral sibutramine dosed to steady state: A PET study using 11C-DASB in healthy humans. J. Cereb. Blood Flow Metab., 24th International Symposium on Cerebral Blood Flow and Metabolism and the 9th International Conference on Quantification of Brain Function with PET. Chicago, IL United States. 29, S65–S66. <https://doi.org/10.1038/jcbfm.2009.125>

Zheng M., Appel L., Luo F., Lane R., Burt D., Risinger R., Antoni G., Cahir M., Keswani S., Hayes W., Bhagwagar Z., 2015. Safety, pharmacokinetic, and positron emission tomography evaluation of serotonin and dopamine transporter occupancy following multiple-dose administration of the triple monoamine reuptake inhibitor BMS-820836. Psychopharmacology 232, 529–540. <https://doi.org/10.1007/s00213-014-3688-x>

**Studies not measuring SERT occupancy of antidepressants (N=23)**

Ananth M.R., DeLorenzo C., Yang J., John Mann J., Parsey R.V., 2018. Decreased pretreatment amygdalae serotonin transporter binding in unipolar depression remitters: A prospective PET study. J. Nucl. Med. 59, 665–670. <https://doi.org/10.2967/jnumed.117.189654>

Berney, A., Nishikawa, M., Benkelfat, C., Debonnel, G., Gobbi, G., Diksic, M., 2008. An index of 5-HT synthesis changes during early antidepressant treatment: alpha-[11C]methyl-L-tryptophan PET study. Neurochem Int 52, 701–708. <https://doi.org/10.1016/j.neuint.2007.08.021>

Cavanagh J., Patterson J., Pimlott S., Dewar D., Eersels J., Dempsey M.F., Wyper D., 2006. Serotonin transporter residual availability during long-term antidepressant therapy does not differentiate responder and nonresponder unipolar patients. Biol. Psychiatry 59, 301–308. <https://doi.org/10.1016/j.biopsych.2005.06.029>

Chou Y.-H., Wang S.-J., Lin C.-L., Mao W.-C., Lee S.-M., Liao M.-H., 2010. Decreased brain serotonin transporter binding in the euthymic state of bipolar I but not bipolar II disorder: A SPECT study. Bipolar Disord. 12, 312–318. <https://doi.org/10.1111/j.1399-5618.2010.00800.x>

Esler, M., Lambert, E., Alvarenga, M., Socratous, F., Richards, J., Barton, D., Pier, C., Brenchley, C., Dawood, T., Hastings, J., Guo, L., Haikerwal, D., Kaye, D., Jennings, G., Kalff, V., Kelly, M., Wiesner, G., Lambert, G., 2007. Increased brain serotonin turnover in panic disorder patients in the absence of a panic attack: reduction by a selective serotonin reuptake inhibitor. Stress 10, 295–304. <https://doi.org/10.1080/10253890701300904>

Goodman M.M., Chen P., Plisson C., Martarello L., Galt J., Votaw J.R., Kilts C.D., Malveaux G., Camp V.M., Shi B., Ely T.D., Howell L., McConathy J., Nemeroff C.B., 2003. Synthesis and characterization of iodine-123 labeled 2beta-carbomethoxy-3beta-(4’-((Z)-2-iodoethenyl)phenyl)nortropane. A ligand for in vivo imaging of serotonin transporters by single-photon-emission tomography. J. Med. Chem. 46, 925–935. <https://doi.org/10.1021/jm0100180>

Gryglewski G., Hahn A., Berroteran-Infante N., Klobl M., Klebermass E.M., Hienert M., Pichler V., Vanicek T., Nics L., Riscka L., Kautzky A., James G.M., Silberbauer L., Hartenbach M., Mitterhauser M., Wadsak W., Hacker M., Kasper S., Lanzenberger R., 2017. Dynamic connectivity analysis of selective serotonin reuptake inhibitor effects, a pharmacological PET/MR study. Eur. Neuropsychopharmacol., 30th European College of Neuropsychopharmacology Congress, ECNP 2017. France. 27, S805–S806.

Huang W., Huang S., Chung T., Huang P., Shen L., Ma K., 2010. Clinical manifestations and I-123 ADAM uptake in different brain areas in patients with major depressive disorder. Eur. J. Nucl. Med. Mol. Imaging, 23rd Annual Congress of the European Association of Nuclear Medicine, EANM 2010. Vienna Austria. 37, S398. <https://doi.org/10.1007/s00259-010-1559-1>

Hummerich R., Reischl G., Ehrlichmann W., Machulla H.-J., Heinz A., Schloss P., 2004. DASB - In vitro binding characteristics on human recombinant monoamine transporters with regard to its potential as positron emission tomography (PET) tracer. J. Neurochem. 90, 1218–1226. <https://doi.org/10.1111/j.1471-4159.2004.02585.x>

James G., Kranz G., Hahn A., Gryglewski G., Hienert M., Spies M., Wadsak W., Mitterhauser M., Kasper S., Lanzenberger R., 2015. Interregional changes in serotonin transporter availability upon treatment with selective serotonin reuptake inhibitors. Eur. Neuropsychopharmacol., 28th European College of Neuropsychopharmacology, ECNP Congress. Amsterdam Netherlands. 25, S327–S328.

Jarkas N., Voll R.J., Williams L., Votaw J.R., Owens M., Goodman M.M., 2008. Synthesis and in vivo evaluation of halogenated N,N-dimethyl-2-(2’- amino-4’-hydroxymethylphenylthio)benzylamine derivatives as PET serotonin transporter ligands. J. Med. Chem. 51, 271–281. <https://doi.org/10.1021/jm0707929>

Kasper S., Tauscher J., Willeit M., Stamenkovic M., Neumeister A., Kufferle B., Barnas C., Stastny J., Praschak-Rieder N., Pezawas L., de Zwaan M., Quiner S., Pirker W., Asenbaum S., Podreka I., Brucke T., 2002. Receptor and transporter imaging studies in schizophrenia, depression, bulimia and Tourette’s disorder--implications for psychopharmacology. World J. Biol. Psychiatry 3, 133–146.

Lee J., Kim B.-H., Kim E., Howes O.D., Cho K.I.K., Yoon Y.B., Kwon J.S., 2018. Higher serotonin transporter availability in early-onset obsessive-compulsive disorder patients undergoing escitalopram treatment: A [11C]DASB PET study. Hum. Psychopharmacol. 33, e2642. <https://doi.org/10.1002/hup.2642>

Leiser S.C., Pehrson A.L., Robichaud P.J., Nielsen K.G.J., Jensen J.B., Smagin G., Song D., Budac D., Frazer A., Sanchez C., 2012. Preclinical studies of the multimodal antidepressant vortioxetine support a potential for improvement of cognitive functions. Neuropsychopharmacology, 51st Annual Meeting of the American College of Neuropsychopharmacology, ACNP 2012. Hollywood, FL United States. 38, S164–S165. <https://doi.org/10.1038/npp.2012.219>

Makkonen I., Riikonen R., Kuikka J.T., Kokki H., Bressler J.P., Marshall C., Kaufmann W.E., 2011. Brain derived neurotrophic factor and serotonin transporter binding as markers of clinical response to fluoxetine therapy in children with autism. J. Pediatr. Neurol. 9, 1–8. <https://doi.org/10.3233/JPN-2010-0446>

Miller J.M., Oquendo M.A., Ogden R.T., Mann J.J., Parsey R.V., 2008. Serotonin transporter binding as a possible predictor of one-year remission in major depressive disorder. J. Psychiatr. Res. 42, 1137–1144. <https://doi.org/10.1016/j.jpsychires.2008.01.012>

Owens M., Dunlop B., Plott S., Zejnelovic F., Craighead W.E., Mayberg H., Nemeroff C., 2014. Estimates of serotonin or norepinephrine transporter occupancy do not predict antidepressant response in a 12 week trial. Neuropsychopharmacology, 53rd Annual Meeting of the American College of Neuropsychopharmacology, ACNP 2014. Phoenix, AZ United States. 39, S460–S461. <https://doi.org/10.1038/npp.2014.281>

Parsey R.V., Ojha A., Ogden R.T., Erlandsson K., Kumar D., Landgrebe M., Van Heertum R., Mann J.J., 2006. Metabolite considerations in the in vivo quantification of serotonin transporters using 11C-DASB and PET in humans. J. Nucl. Med. 47, 1796–1802.

Rischka L., Hahn A., Gryglewski G., Philippe C., Nics L., Hartenbach M., Traub-Weidinger T., Mitterhauser M., Wadsak W., Hacker M., Kasper S., Lanzenberger R., 2016. Impact of attenuation correction in hybrid PET/MR imaging of serotonin transporter occupancy. Eur. Neuropsychopharmacol., 29th European College of Neuropsychopharmacology Congress, ECNP 2016. Austria. 26, S303.

Smith, D.F., Stork, B.S., Wegener, G., Jakobsen, S., Bender, D., Audrain, H., Jensen, S.B., Hansen, S.B., Rodell, A., Rosenberg, R., 2007. Receptor occupancy of mirtazapine determined by PET in healthy volunteers. Psychopharmacology (Berl) 195, 131–138. <https://doi.org/10.1007/s00213-007-0877-x>

Smith G.S., Lotrich F.E., Malhotra A.K., Lee A.T., Ma Y., Kramer E., Gregersen P.K., Eidelberg D., Pollock B.G., 2004. Effects of serotonin transporter promoter polymorphisms on serotonin function. Neuropsychopharmacology 29, 2226–2234. <https://doi.org/10.1038/sj.npp.1300552>

Stein, D.J., van Heerden, B., Hugo, C., van Kradenburg, J., Warwick, J., Zungu-Dirwayi, N., Seedat, S., 2002. Functional brain imaging and pharmacotherapy in trichotillomania. Single photon emission computed tomography before and after treatment with the selective serotonin reuptake inhibitor citalopram. Prog Neuropsychopharmacol Biol Psychiatry 26, 885–890.

Wilson, A.A., Ginovart, N., Hussey, D., Meyer, J., Houle, S., 2002a. In vitro and in vivo characterisation of [11C]-DASB: a probe for in vivo measurements of the serotonin transporter by positron emission tomography. Nucl Med Biol 29, 509–515.

**Review articles (N=5)**

Dreimuller N., Tadic A., Grunder G., Hiemke C., 2011. How much citalopram is equivalent to 50 ng/ml sertraline? Standardization of serum concentrations of six different SSRIs by measuring serotonin transporter occupancy. Pharmacopsychiatry, 27th Symposium of the AGNP. Munich Germany. 21. <https://doi.org/10.1055/s-0031-1292468>

Grunder G., 2016. PET imaging for guiding definition of therapeutic reference ranges-new drugs, new guidelines. Pharmacopsychiatry, 12th Symposium of the AGNP Task Force on Therapeutic Drug Monitoring in Psychiatry. Germany. 26. <https://doi.org/10.1055/s-0036-1582031>

Meyer, J.H., 2007. Imaging the serotonin transporter during major depressive disorder and antidepressant treatment. J Psychiatry Neurosci 32, 86–102.

Saulin A., Savli M., Lanzenberger R., 2012. Serotonin and molecular neuroimaging in humans using PET. Amino Acids 42, 2039–2057. <https://doi.org/10.1007/s00726-011-1078-9>

Zipursky, R.B., Meyer, J.H., Verhoeff, N.P., 2007. PET and SPECT imaging in psychiatric disorders. Can J Psychiatry 52, 146–157. <https://doi.org/10.1177/070674370705200303>

**Studies not providing occupancy data in means and standard deviations (N=4)**

Berding G., Wilke F., Bokemeyer S., Haense C., Geworski L., Bengel F., Muller-Vahl K., 2013. Evidence for an involvement and therapeutic relevance of the serotonergic system in Tourette syndrome with co-morbiditie. Eur. J. Nucl. Med. Mol. Imaging, 26th Annual Congress of the European Association of Nuclear Medicine, EANM 2013. Lyon France. 40, S218. <https://doi.org/10.1007/s00259-013-2535-3>

Erlandsson K., Sivananthan T., Lui D., Spezzi A., Townsend C.E., Mu S., Lucas R., Warrington S., Ell P.J., 2005. Measuring SSRI occupancy of SERT using the novel tracer [ 123I]ADAM: A SPECT validation study. Eur. J. Nucl. Med. Mol. Imaging 32, 1329–1336. <https://doi.org/10.1007/s00259-005-1912-y>

Herold N., Uebelhack K., Franke L., Amthauer H., Luedemann L., Bruhn H., Felix R., Uebelhack R., Plotkin M., 2006. Imaging of serotonin transporters and its blockade by citalopram in patients with major depression using a novel SPECT ligand [123I]-ADAM. J. Neural Transm. 113, 659–670. <https://doi.org/10.1007/s00702-005-0429-7>

Parsey R.V., Kent J.M., Oquendo M.A., Richards M.C., Pratap M., Cooper T.B., Arango V., Mann J.J., 2006. Acute Occupancy of Brain Serotonin Transporter by Sertraline as Measured by [11C]DASB and Positron Emission Tomography. Biol. Psychiatry 59, 821–828. <https://doi.org/10.1016/j.biopsych.2005.08.010>

**Supplementary Table 2.** Parameter estimates for the 2-parameter Michaelis-Menten models employed to fit individual curves in Figure 1

| **Antidepressant** | **RoI** | **V_m_** | **K** |
| --- | --- | --- | --- |
| Citalopram | Striatum | 83.98 | 2.33 |
| Desvenlafaxine | Amygdala | 106.43 | 11.57 |
| Desvenlafaxine | Midbrain | 99.46 | 10.92 |
| Desvenlafaxine | Striatum | 100.22 | 16.11 |
| Desvenlafaxine | Thalamus | 100.11 | 21.17 |
| Duloxetine | Thalamus | 90.75 | 6.27 |
| Escitalopram | Caudate | 75.35 | 0.66 |
| Escitalopram | Dorsal Raphe Nucleus | 89.57 | 2.72 |
| Escitalopram | Putamen | 69.32 | 1.22 |
| Escitalopram | Thalamus | 78.67 | 2.10 |
| Fluoxetine | Striatum | 86.12 | 1.89 |
| Paroxetine | Striatum | 97.14 | 5.60 |
| Sertraline | Striatum | 92.01 | 7.72 |
| Venlafaxine XR | Striatum | 90.07 | 5.80 |
| Vortioxetine | Raphe Nuclei | 102.24 | 4.68 |

RoI: brain region of interest; Vm: the horizontal asymptote (expressing maximum occupancy); K: the dose where the occupancy is halfway between 0 and V_m_.
